# Notch3 signalling and vascular remodelling in pulmonary arterial hypertension

**DOI:** 10.1042/CS20190835

**Published:** 2019-12-23

**Authors:** Hannah E. Morris, Karla B. Neves, Augusto C. Montezano, Margaret R. MacLean, Rhian M. Touyz

**Affiliations:** 1 Institute of Cardiovascular and Medical Sciences, University of Glasgow, Glasgow, Scotland; 2 Strathclyde Institute of Pharmacy and Biomedical Sciences, University of Strathclyde, Glasgow, Scotland

**Keywords:** Hes, Hey, Notch receptors, pulmonary arteries, signal transduction, vascular smooth muscle cells

## Abstract

Notch signalling is critically involved in vascular morphogenesis and function. Four Notch isoforms (Notch1–4) regulating diverse cellular processes have been identified. Of these, Notch3 is expressed almost exclusively in vascular smooth muscle cells (VSMCs), where it is critically involved in vascular development and differentiation. Under pathological conditions, Notch3 regulates VSMC switching between the contractile and synthetic phenotypes. Abnormal Notch3 signalling plays an important role in vascular remodelling, a hallmark of several cardiovascular diseases, including pulmonary arterial hypertension (PAH). Because of the importance of Notch3 in VSMC (de)differentiation, Notch3 has been implicated in the pathophysiology of pulmonary vascular remodelling in PAH. Here we review the current literature on the role of Notch in VSMC function with a focus on Notch3 signalling in pulmonary artery VSMCs, and discuss potential implications in pulmonary artery remodelling in PAH.

## Introduction

Notch proteins are cell membrane receptors that mediate signalling between cells and hence play an important role in cell-to-cell communication [1]. Four Notch receptors and at least five ligands have been identified in mammals [2,3] and while all Notch receptors have the same primary structure, they mediate diverse cellular effects. Notch isoforms are ubiquitous and expressed in a cell-specific manner, with Notch3 being present almost exclusively in vascular smooth muscle cells (VSMCs), where it controls maintenance of cell phenotype and growth [4]. Notch signalling induces transcription of target genes that influence cell differentiation, maturation, proliferation and apoptosis and is critically involved in the regulation of VSMC phenotypic switching (contractile-to-proliferative) [5]. In the vascular system the Notch pathway is involved in vascular development, angiogenesis and arteriovenous specification [6]. Given the importance of Notch3 in the regulation of VSMC function, it is not surprising that abnormal Notch3 signalling has been implicated in cardiovascular diseases associated with excessive VSMCs proliferation and vascular remodelling, including pulmonary arterial hypertension (PAH).

The hallmark of PAH is vascular dysfunction and structural remodelling of small pulmonary arteries, leading to thickening of the vascular media and luminal occlusion due to uncontrolled proliferation, cytoskeletal disorganisation and dedifferentiation of VSMCS [7]. This vasculopathy of small pulmonary arteries leads to increased pulmonary artery pressure and right ventricular failure, often leading to death [8]. Multiple processes contribute to the pulmonary artery phenotype in PAH including genetic factors, DNA damage, microRNAs (miRs), sex hormones, oxidative stress and altered cell metabolism [9,10]. In addition, Notch3 has been implicated as a potential crucial mediator of VSMC dedifferentiation and proliferation in pulmonary arteries in PAH [11]. This review discusses Notch in the regulation of VSMCs function with a focus on Notch3 in pulmonary VSMCs and highlights recent advances on the role of Notch3 in pulmonary artery remodelling in PAH.

## Notch signalling in the vasculature

### The Notch family

The Notch signalling pathway is a highly conserved system that regulates cell fate determination and differentiation [1]. Four *NOTCH* gene family paralogs have been described in mammals encoding Notch receptors 1, 2, 3 and 4. Mature Notch receptors share the same structural features consisting of a Notch extracellular domain (NECD) associated non-covalently with a Notch intracellular domain (NICD) [2], creating a single-pass heterodimeric transmembrane receptor. Multiple ligands bind to Notch including members of the Jagged protein (Jag 1, 2) and Delta-like protein (Dll 1, 3, 4) families [3]. Ligand binding triggers the NICD to detach and translocate into the nucleus to interact with downstream targets. Accordingly, Notch proteins act both as transmembrane receptors as well as nuclear transcription factors transmitting signals from the plasma membrane to the nucleus without involvement of second messengers [12].

### Canonical and non-canonical signalling through Notch

**Figure 1:**
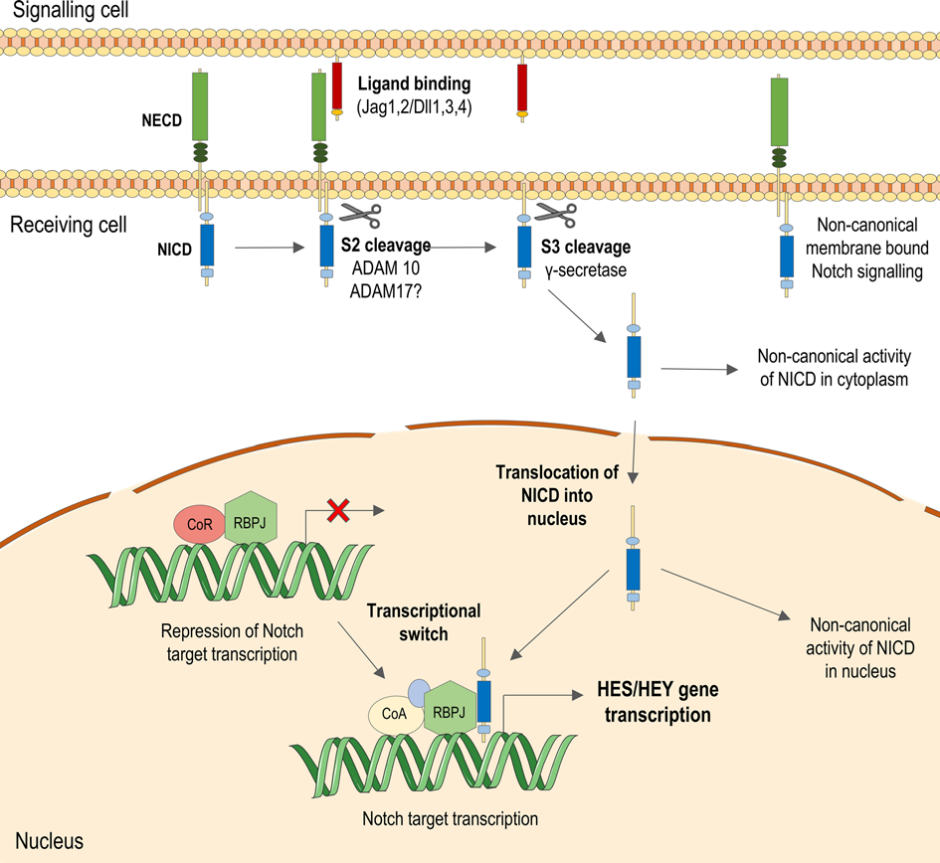
The canonical Notch signalling pathway In the canonical Notch pathway, a ligand physically interacts with and binds to the NECD on an adjacent cell. This induces cleavage events by two sheddases, ADAM10 and γ-secretase, allowing cleaved NICD to translocate to the nucleus. In the nucleus, the NICD binds to recombining binding protein suppressor of hairless (RBP-Jκ) and recruits co-activators including Mastermind-like (MAML), which stabilises the interaction between the NICD and RBP-Jκ. This complex promotes transcription of Notch target HES and HEY genes. These transcription factors then affect expression of many more genes involved in processes such as cell cycle progression, survival, and cellular phenotype. Non-canonical Notch signalling pathways are also indicated.

Notch signal transduction is relayed via cell–cell interaction, where cell-bound Jagged or Delta-like ligands interact with EGF-like repeats in the NECD of Notch on an adjacent cell (Figure 1). This mechanism therefore only occurs between cells physically close to each other, limiting the range of signalling. The core canonical Notch signal pathway following ligand binding is dependent on a series of proteolytic processes [3]. The most functionally important are cleavage events occurring at site 2 (S2) and site 3 (S3) of Notch. S2 cleavage occurs upon ligand binding when an ADAM (A Disintegrin And Metalloproteinase) protease mediates shedding of the NECD from the remainder of the receptor. ADAM10 is generally described as the main sheddase involved in S2 cleavage. However a related sheddase, ADAM17/TNFα-converting enzyme (TACE), can also cleave Notch [13,14] with partial redundancy between these two enzymes [15]. S3 cleavage by the γ-secretase enzyme then releases the NICD from the membrane into the cytoplasm of the cell receiving the signal [16]. Cleaved NICD translocates to the nucleus to form a complex with the nuclear RBP-Jκ (Recombination Signal Binding Protein For Immunoglobulin κ J Region) repressor protein (known as Su(H) in flies and as LAG-1 in worms).

In the absence of NICD, transcription of Notch effector genes is repressed by RBP-Jκ but when the NICD and co-activators such as the Mastermind-like protein bind this complex [17], it becomes a transcriptional activator of the two major Notch target gene families: *HES* and *HEY* [3]. The seven *HES* members and three *HEY* members encode basic helix–loop–helix (bHLH) transcription factors that act as repressors of transcription, thereby influencing expression of numerous downstream gene targets. Additionally, while Hairy and enhancer of split (Hes) and Hairy/enhancer-of-split related with YRPW motif (Hey) are the major transcriptional targets induced through Notch activation, Notch signalling induces expression of other genes such as c-Myc [18], cyclins [19,20] and platelet-derived growth factor receptor β [21]. Notch signalling is highly versatile depending on the ligand–receptor interaction and cell-specific expression of particular Notch isoforms [22,23].

Non-canonical Notch signalling has also been described where signalling occurs independent of ligand/transcription. Examples include interaction of the NICD with proteins in the cytoplasm [24,25], with non-Notch targets in the nucleus [26], and also where downstream Notch-associated signalling is induced independently of Notch receptor activation [27,28], or independently of RBP-Jκ activation [29]. However, non-canonical Notch signalling is not well characterised, particularly in the vasculature.

### Differential Notch dynamics and functions

Another factor adding to the diversity of Notch-mediated signalling is differential dynamics of Notch ligands, receptors and effectors. For example, within certain tissues ligands Dll1 and Dll4 can have interchangeable functions [30] while in others the two effectors have opposing actions [31]. This may partly be explained by ligand affinities to Notch. The extracellular domain of Dll4 has over ten-fold the affinity for Notch1 than Dll1 [32], which could lead to differences in their signalling effects. Dll1 and 4 also signall through Notch1 with different dynamics; Dll1-stimulated activation is pulsatile while Dll4-stimulated is sustained [31], which can differentially influence downstream effects. Notch activation also exhibits ligand preference-associated lineage discrimination. In embryonic stem cells Jag1 stimulation favours commitment to differentiation into neural cells, while Dll4 favours differentiation into mesoderm [33].

Similarly, there are differences in the way transcription of Hes and Hey Notch effectors are induced. Expression of *Hes1* and *5* [34,35], *Hes7* [36], and all three *Hey* genes [37,38] are directly induced by activation of the classical Notch pathway. However, *Hes2, Hes3* [35] and *Hes6* [38,39] are induced independent of signalling through Notch. In certain tissues ligands acting through the same receptor can activate different downstream gene targets. Co-culture of Dll4-expressing cells with C2C12 cells overexpressing Notch1 produced approximately three- to five-fold greater expression of *Hey1* and *HeyL* than Dll1-expressing cells could [31]. Also, duration and dynamics of Notch activation can potentially influence distinct *Hes/Hey* expression patterns. *Hes1* appears to respond even to short pulsatile Notch dynamics, while *Hey1* and *HeyL* require sustained Notch activation in order for their expression to be induced [31]. Finally, the NICD may also show preference for transcription of certain Notch effector gene, for example Notch3 ICD (NICD3) contains a transactivation domain for interaction with the HES5 promoter [40].

### Notch in vascular development

Notch signalling is key for normal angiogenesis and vasculogenesis in the developing mammal embryo. However, each receptor appears to contribute differently. While knockout of Notch1 [41] or targeted mutagenesis of Notch2 [42] and Notch1/Notch4 [43] produce an embryonic lethal phenotype in mice, knockout of Notch3 does not affect embryo viability [44] but does affect vascular structure.

**Figure 2:**
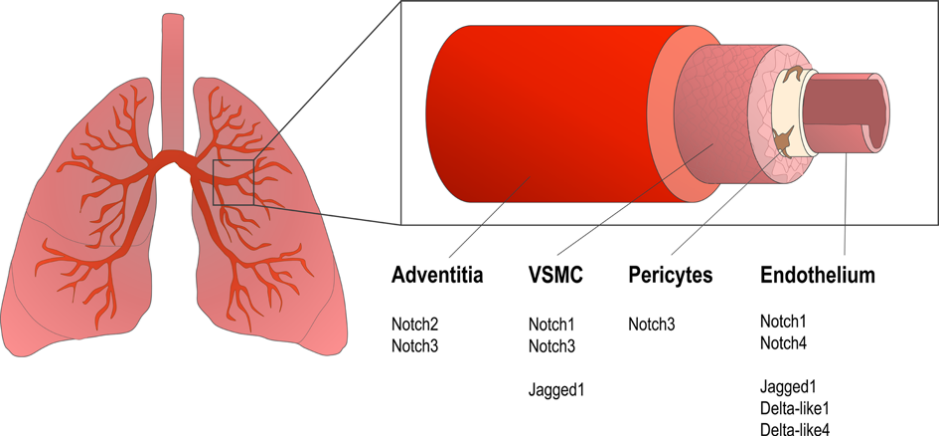
Notch receptors and ligands in the pulmonary vasculature All four Notch receptor isoforms are expressed in the pulmonary circulation. Notch ligands are predominantly expressed in endothelial cells, with weak expression elsewhere. Notch3 expression is focussed in the medial layer during development and in the adult, its expression is more abundant in smaller diameter arteries and arterioles where it appear to have the most influence on remodelling processes.

All four Notch receptors are expressed in the developing mouse lung, but there is differential expression of each receptor type in different vascular cells (Figure 2). Notch1 transcripts are expressed in both vascular epithelial structures, including the endothelium and non-vascular epithelial structures [45], and also in pulmonary artery smooth muscle cells (PASMCs) [21]. Notch2 is expressed in the mesenchyme, where it is abundant in the adventitia of pulmonary arteries [46], while Notch3 is expressed in PASMCs [46] and pericytes. Notch4 has been suggested as an endothelial-specific Notch in the pulmonary vasculature [47], as is suggested to be the case in other vascular beds [47,48]. In adult human pulmonary vessels Notch3 expression seems limited to mural cells (VSMCs and pericytes) and fibroblasts, and expression is concentrated in distal pulmonary arteries [11].

Little is known about Notch ligand expression specific to the pulmonary vasculature. Both Dll1 [49] and Dll4 [50] are expressed during embryogenesis in endothelial cells of mouse lung. Jag1 is expressed in the endothelium of pulmonary vessels during development and after birth [45] and is also present in PASMCs [46]. There is also a lack of expression profiling of Notch effectors Hes and Hey in pulmonary arteries. *In silico* studies of human vascular expression profiles of downstream Notch effectors report ubiquitous expression of *HES1, HEY1* and *HEY2* genes in endothelial cells [38]. Expression of all *HEY* family members is reported in embryonic arteries with strong *HEYL* expression in the developing pulmonary artery [51]. Hey2 protein has also been proposed as a cell type-specific primary target of Notch that can be up-regulated by ligand interaction in VSMC but not in other contractile cells [52].

### Notch and regulation of the VSMC phenotype

VSMC phenotype is tightly regulated by Notch3. In *Notch3^−/−^* mice, cerebral and tail arteries exhibit a thinner vascular medial layer with altered morphology of VSMCs. In these mice from birth to postnatal day 28, normal maturation processes are significantly impaired, and arteries follow a maturation pattern more closely resembling veins rather than arteries [6]. These alterations are more noticeable in smaller resistance arteries than in elastic arteries in line with the greater abundance of Notch3 in small vessels compared with large vessels. Moreover, expression and localisation of smoothelin, a marker of mature VSMCs, is reduced in *Notch3^*−/−*^* arteries [6] suggesting impairment of VSMC differentiation and maturation.

Similar findings were observed in the pulmonary vasculature, using a conditional Notch3 knockout mouse modell. In the early postnatal period, arteries were misshapen, non-cohesive and had a reduction in expression and organisation of α-smooth muscle actin (SMA) in the medial layer [46]. These morphological changes were associated with reduced expression of several smooth muscle-related genes, including α- and γ-SMA, smooth muscle protein 22-α (SM22α), smoothelin, calponin and smooth muscle myosin heavy chain (SM-MyHC) in *Notch3^*−/−*^* lung, in the embryo and adulthood [46]. Findings from these studies indicate Notch3 is not only involved in arterial development but also in the vascular maturation process especially post-natally.

Maintenance of the VSMC phenotype requires ligand-induced Notch signalling, where VSMC Notch is activated by cell–cell contact with Notch ligands expressed by neighbouring endothelial cells. Co-culture of human coronary VSMCs and endothelial cells, which express Notch ligand Jag1, increase expression of *HEYL* and *HES1* in VSMCs [53]. These conditions also induce α-SMA, SM22α and calponin gene expression, which are reduced by down-regulation of Jag1 in endothelial cells [53,54]. Co-culture of VSMCs with endothelial cells also increases expression of Notch3, an effect that is inhibited by Jag1-deficient endothelial cells indicating auto-regulation of Notch in vascular cells [53].

### Notch3 in VSMC dedifferentiation from a contractile to a synthetic phenotype

While VSMCs are characteristically contractile cells, they undergo phenotypic changes, especially in pathological conditions [55]. They are highly plastic and capable of phenotypic switching promoting proliferation, migration and growth, important processes that underlie vascular remodelling. This dedifferentiation process in which the VSMC phenotype becomes more synthetic is also crucial for vascular healing. Because of this synthetic phenotypic switching is often associated with vascular pathologies in which there is an element of vascular injury, such as atherosclerosis, neoinitimal formation and restenosis [56], systemic hypertension [57], and PH [58].

Jag1 stimulation of Notch signalling in 10T1/2 fibroblasts, a model of myofibroblast/smooth muscle cell differentiation, leads to repression of myocardin-induced expression of SMα-actin, SM-MyHC, and SM22α, most likely through Hey1 transcription, and hence represses the contractile phenotype [59]. Similarly, overexpression of NICD3 in 10T1/2 cells inhibits expression of smooth muscle-actin, SM22, and calponin in the presence of TGF-1, an inducer of fibroblast differentiation towards a smooth muscle cell fate [60].

Overexpression of constitutively active NICD1 and NICD3 and activation of Notch3 signalling in human VSMCs down-regulates expression of smooth muscle markers including α-actin, calponin, myosin, and smoothelin [61]. This evidence, together with findings from knockout models, suggests that Notch3 plays an important role in regulating contractile and synthetic genes in vascular cells, ultimately impacting cell phenotype and fate. Many molecular processes underlie Notch3-mediated phenotypic switching including modulation of Krüppel-like factors (KLF), a family of transcription factors that also contributes to vascular cell homoeostasis. Under physiological conditions expression of KLF4 and KLf5 is low, but in the context of vascular injury expression is markedly increased [62,63]. KLF4 has a negative effect on cell cycle progression and can also induce expression of smooth muscle cell genes promoting a contractile VSMC phenotype [64]. Notch3 down-regulates expression and activity of KLF4 in the gastrointestinal tract [65,66] and promotes cell proliferation [66]. Notch signalling can also regulate expression of KLF5 which, unlike KLF4, causes VSMC proliferation and a synthetic phenotype, important in vascular remodelling in PAH, particularly in hypoxia [67,68]. Hence Notch and KLFs are closely linked, but mechanisms of interaction in VSMCs remain unclear.

Dysregulated Notch3 signalling promotes VSMC growth [69]. Constitutive NICD3 expression induced by viral transfection produces a significant increase in VSMC number [70]. Adenoviral NICD3 overexpression in rat PASMCs induces a significant increase in proliferation accompanied by up-regulation of Hes1 transcription factor expression and down-regulation of p27_Kip1_ cyclin inhibitor protein. The reduction in p27_Kip1_ is inhibited by Hes1 siRNA [71]. This is also observed in human PASMCs where cell number and growth curves are increased in hPASMCs overexpressing NICD3 [11]. A concomitant increase in downstream Notch effector Hes5 was also reported. The role of Hes5 was confirmed by siRNA-mediated knockdown of Hes5 in NICD3 overexpressing PASMCs, which limits the proliferation rate to levels comparable with control [11]. Hes transcription factors may be important transcriptional targets of Notch3 signalling in PASMCs.

Notch3 activation also mediates vascular hypertrophy by decreasing VSMC apoptosis. Constitutive NICD3 overexpression in rat VSMCs confers resistance to apoptosis induced by Fas ligand [72] in response to serum deprivation [70] and to UV-B irradiation [73]. Evidence from these studies suggest that Notch3 decreases the activity of the apoptosis promoter caspase-3, [70,73] and promotes MAPK-mediated cell survival by increasing expression of pro-survival genes for Bcl2, survivin and c-FLIP [73] in line with evidence from Notch3 knockout mice. Conversely, aortic VSMCs from *Notch3^−/−^* mice exhibit reduced expression of mRNA for pro-survival genes, combined with an increased cleavage and activity of caspase-3 in response to serum challenge. In UV-treated aortic VSMCs overexpressing NICD3, siRNA down-regulation of Notch3 mirrors the effects of genetic deletion of Notch3 in knockout mice [73].

While Notch3 is typically associated with VSMC growth, it also plays a role in regulating vascular contraction. This is supported by studies in patients with Notch3 mutations [Cerebral Autosomal Dominant Arteriopathy with Subcortical Infarcts and Leukoencephalopathy (CADASIL)] and transgenic mice with Nox3 deficiency, where cerebral and peripheral arteries exhibit not only structural remodelling but also functional changes including endothelial dysfunction and impaired myogenic tone [74]. Pharmacological inhibition of Notch cleavage decreases pulmonary arterial pressure in isolated mouse lungs, further indicating a role for Notch3 in the regulation of vascular contraction [75].

## Notch3 signalling in pathological vascular remodelling-implication in PAH

Since Notch3 signalling is a key regulator of vascular function and because it has a VSMC-specific expression pattern, it is not surprising that it has been considered as a candidate target in conditions involving pathological remodelling of blood vessels, including PAH.

Pulmonary hypertension (PH) comprises a group of rare and progressive conditions that affect the small arteries of the pulmonary vasculature leading to right ventricular failure. Clinically PAH is described as a persistently elevated pulmonary arterial pressure of ≥25 mmHg and symptoms including fatigue, exercise-induced dyspnea, shortness of breath and oedema [76]. However, a combination of non-specific symptoms and the requirement of invasive methods to confirm a diagnosis means that PAH is often diagnosed at a late stage and subsequently has a poor prognosis. In addition, the current lack of understanding of the underlying molecular mechanisms involved in PAH pathogenesis means that no cure is available, and treatments are often suboptimal, making PAH a fatal disease with an estimated transplant-free survival rate at 3 years after diagnosis of only ∼71% [8].

**Figure 3:**
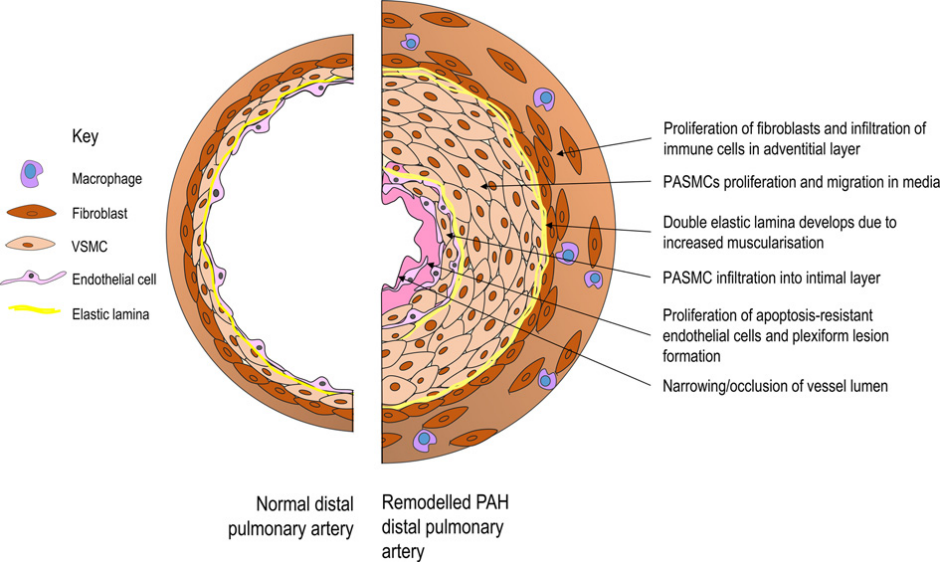
Pulmonary vascular remodelling in PAH Diagram shows a small pulmonary artery in cross-section. Vascular cells in all three layers of the vessel, the intima, media and adventitia, contribute to vessel remodelling in PAH. The result of the pulmonary vascular remodelling is narrowing or complete obstruction of the vessel lumen, further raising blood pressure in the generally low-pressure pulmonary circulation and increasing the load on the right ventricle.

PAH is characterised by vasoconstriction, pathological maladaptive vascular remodelling, and subsequent right heart hypertrophy due to increased resistance in the pulmonary circulation (Figure 3). PAH affects the smaller distal arteries of the pulmonary circulation and is heritable, due to a genetic mutation, or idiopathic where the underlying cause is unknown. Vascular cells, in particular PASMCs in the medial layer of pulmonary arteries, display a pro-proliferative and anti-apoptotic/pro-survival phenotype, which underlies the extensive remodelling in severe PAH [7]. Numerous systems have been implicated in these changes, including growth factors, cytokines, metabolic factors, sex hormones and BMP signalling among others. More recently, activation of Notch3 has been suggested as an important mediator of vascular remodelling and dysfunction in PAH [11].

### Notch3 and PH in experimental models

Li et al. [11] were among the first to explore the role of Notch3 in PAH by studying the development of hypoxia-induced PAH in *Notch3^−/^*^−^ mice. Compared with wild-type and *Notch3^+/−^* mice, Notch3 knockout mice did not develop PH in response to 6 weeks of chronic hypoxia [11]. Histologically, *Notch3^−/^^−^* mice failed to show muscularisation of the small pulmonary arteries and arterioles with no hypertrophic remodelling and VSMC proliferation compared with wild-type and heterozygous mice [11]. Reduced muscularisation and remodelling in *Notch3^−/^^−^* mice resulted in maintenance of diffuse vascular blush in the distal pulmonary tree in knockout animals, while significant vascular pruning was observed in lungs from hypoxic control mice [11].

### Notch3 expression in PAH

Expression of Notch1, Notch3, Jagged1 and Herp2 (Hey1) are increased in lungs from mice with hypoxia-induced PAH compared with normoxic animals [77]. Significantly elevated Notch3 mRNA and protein levels have also been confirmed in lungs from monocrotaline (MCT)-treated rats compared with control rats [11,77,78]. In addition, expression of Notch3 (gene and protein) is increased in the Sugen-hypoxic rat [79], which recapitulates the human condition.

These findings in experimental models of PAH have also been observed in patients with PAH. Increased expression of Notch3 signalling pathway components has been demonstrated in pulmonary VSMCs from patients with PAH and the severity of PAH correlated with the amount of Notch3 protein in the lung in humans and rodents [11]. In particular, significantly higher levels of Notch3 mRNA and NICD3 protein are present in hPASMCs from idiopathic PAH patients compared with the low levels in control lungs [11]. This coincides with a concurrent decrease in smoothelin and MHC smooth muscle marker expression, and a significantly faster growth rate in PAH cells compared with non-PAH cells [11]. These findings suggest an association between NICD3 overexpression, PASMC dedifferentiation and increased PASMC proliferation in PAH.

### Notch3 mutations in PAH

While there is little evidence that Notch3 mutations cause PAH, Chida et al. [80] identified two novel missense mutations (G840E and T900P) of the *NOTCH3* gene in two cases of childhood idiopathic PAH. Both mutations affected residues in the highly conserved EGF-like repeats of the Notch3 receptor, the region in which the vast majority of CADASIL mutations also occur and ligand binding occurs. *In vitro* studies using cells expressing these mutant Notch3 receptors, revealed increased cell viability and proliferation suggesting that these mutations have a gain-of-function action on Notch3 signalling [80]. More recently, another group also identified a synonymous single nucleotide polymorphism (SNP) in NOTCH3 (A2146A) as significantly associated with persistant PH of the newborn in a Chinese cohort, with detection in three patients [81].

### A role for the Hes family in PAH

It is likely some of the heterogeneity in Notch signalling effects is due to differential and potentially cell type-specific effects of Notch ligands and effectors in different tissues. In terms of downstream Notch effectors driving the effects of Notch3 in PAH, Hes family members seem to be most important in the pulmonary vasculature. In PASMCs from MCT-treated rats increased NICD3 expression was associated with up-regulation of Hes1 and a concurrent decrease in p27_Kip1_ expression, thereby reducing slowing of the cell cycle [11]. Hes5 has also been implicated in Notch3-induced PASMC proliferation in PAH. This is particularly significant, given the reported ‘preference’ of Notch3 for inducing Hes5 [40]. As with Notch3, Hes5 expression has been seen specifically localised to the PASMCs from small pulmonary arteries in both human and rat lung [11]. Following Hes5 knockdown with siRNA, PASMCs from patients with and without PAH demonstrated a reduction in cell proliferation, an effect that was more significant in PAH VSMCs [11]. Hes5 siRNA treatment also increased expression of genes for myosin heavy chain 11 and smoothelin, suggesting that inactivation of Hes5-mediated signalling in PAH cells promotes phenotypic switching from a synthetic to a more contractile phenotype [11]. Notch3-Hes5 mRNA expression is also increased in MCT-induced PH in rats, and found to positively correlate with mean pulmonary arterial pressure [82].

### Notch3 and hypoxia in PASMCs

**Figure 4:**
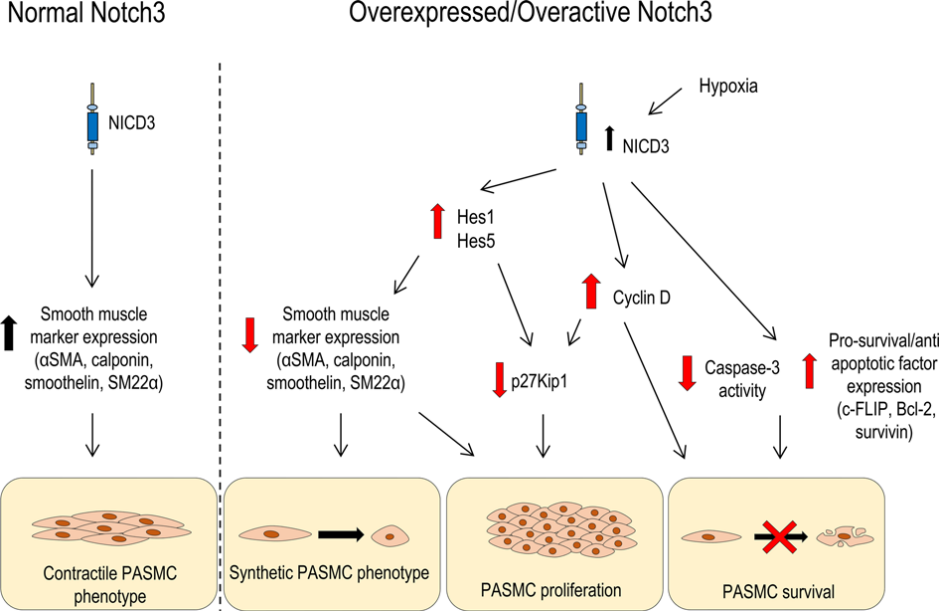
An overview of proposed mechanisms whereby Notch3 signalling influences PASMC function, in the context of normal and overactive Notch3 signalling Under normal conditions, Notch3 is an important regulator of the contractile phenotype in VSMCs. Under conditions of increased Notch3/Notch3 activity, VSMCs undergo changes promoting a synthetic, proliferative and pro-survival phenotype in PASMCs.

Hypoxia, a major factor in vascular remodeling in PAH, is an important regulator of Notch3-mediated differentiation of VSMCs (Figure 4). Hypoxia inhibits differentiation of the C2C12 immortalised mouse myogenic cell line, and this effect was abrogated when cells were treated with hypoxia in the presence of γ-secretase inhibitor (GSI) L-685,458, which prevents Notch cleavage [83]. In the lungs, hypoxia is especially relevant, both *in utero* where hypoxic vasoconstriction of pulmonary arteries allows the fetal circulation to bypass the lungs, and as a means of preserving ventilation/perfusion matching after birth. *In vitro*, human PASMCs from healthy individuals exposed to hypoxic air (3% O_2_) proliferate at a higher rate compared with normoxic cells [84]. *In vivo*, chronic hypoxia is a well-characterised promoter of PH as it induces pulmonary vascular remodeling by inducing expression of hypoxia inducible factor 1 (HIF-1α), a master regulator of cellular response to hypoxia. Notch3 has been identified as a downstream target of hypoxia, which is up-regulated through HIF-1α. Hypoxia significantly increases the number of PCNA-positive PASMCs in lungs from *Notch3^+/+^* mice, but this effect is less apparent in PASMCs from *Notch3^−/−^* mice lungs [11]. This suggests hypoxia is a mediator of proliferation in PASMCs via up-regulation of Notch3 signalling, a mechanism absent from Notch3-deficient mice.

Exposure of neonatal mouse pups to chronic hypoxia (13% O_2_) to induce PH significantly increases pulmonary expression of Notch3 and Notch-related genes including *Hes5, Jag1* and *ADAM17*, as well as up-regulating expression of anti-apoptotic *BCL2L1* (Bcl-xL), and cell cycle promoter *CCND1* (Cyclin D) [85]. A similar up-regulation of Notch signalling was observed in chronically hypoxic adult mice, with an increase in lung NICD3 protein that positively correlated with the number of weeks spent in hypoxia and HIF-1α [84]. This further suggests that Notch3 signalling is activated in chronic hypoxic conditions through HIF-1α with potential to contribute to remodelling in the distal pulmonary circulation.

### The cancer theory of PAH and Notch3

PAH cells share many characteristics with cancer cells, namely a propensity for survival and proliferation under unfavourable, hypoxic conditions, and the involvement of many of the same signalling pathways such as mammalian target of rapamycin (mTOR), HIPPO and growth factor signalling [86]. Additionally, Notch3 up-regulation and overactivation have been demonstrated in various cancers, particularly ovarian [87–89] and non-small cell lung cancers [90–92].

An important feature of cancer is an increase in DNA damage. This is also seen in human PAH cells and distal PAs from PAH rodent models and suggested as a response to inflammation and oxidative stress [93] and increased sensitivity to mutagens [94]. In established PAH this is associated with maladaptive sustained increase in DNA repair machinery in PAH cells as they cope with DNA damage [93] promoting a pro-survival, pro-proliferative response in the pulmonary vascular cells leading to pulmonary artery occlusion and increased pulmonary pressure [95]. An increase in proteins which sense DNA damage and/or promote repair, is seen in PAH patient cells and models, and may contribute to a cancer cell-like phenotype.

Among these are translationally controlled tumour protein (TCTP) that senses and repairs DNA damage to maintain genomic integrity. This includes poly (ADP-ribose) polymerase 1 (PARP-1), a key first responder in the sensing and reparation of DNA strand breaks, and mTOR. All are up-regulated in established PAH. TCTP is implicated as a novel mediator of endothelial survival in hPAH [96], and potential PAH biomarker [97]. Additionally, PARP-1 inhibition *in vivo* can reverse PAH in two models [93] and PARP-1 inhibitor Olaparib is now being explored in clinical trials as a potential PAH therapy following success in cancer trials (ClinicalTrials.gov identifier NCT03251872). mTOR regulates cell survival/death through modulation of cell growth and autophagy, and mTOR complex 1 (mTORC1) is a major pathway employed by cells to deal with stresses and DNA damage [98]. Increased mTORC1 and mTORC2 expression [99], and mTOR phosphorylation [100,101] have been observed in PAH patient lung vascular tissue and cells. mTORC1 inhibitor rapamycin prevents hypoxia and HIF-1α induced increase in PASMC proliferation *in vitro* [99] and *in vivo* [99,102]. Knockout of mTOR or mTORC1 regulatory protein Raptor in smooth muscle also attenuates HPH development in mice [103]. mTORC2 also promotes PASMC survival; siRNA for mTORC2 component Rictor decreased proliferation and induced apoptosis in IPAH PASMCs [99], suggesting involvement of mTORC2 in the cancer-like PAH phenotype. Additionally, the substrate of mTORC2, Akt, can then activate mTORC1 [104].

The pro-proliferative and pro-survival mTOR pathway has also been shown to interact with Notch3 in the context of PAH. mTOR has previously been identified as a positive regulator of Notch3 [105]. Following chronic hypoxia both mTOR and Notch3 signalling are up-regulated in the lung, and increased Notch3 cleavage was prevented by treatment with mTOR inhibitors rapamycin [84] and temsirolimus [85], suggesting that Notch3 signalling is downstream of mTOR. NICD interacts with mTOR-Rictor in a non-canonical manner, independent of RBP-Jκ binding, to block apoptosis through an NIC-mTORC2-Akt pro-survival signalling cascade [106]. Functionally, when PARP-1 is activated by DNA damage, phosphoinositide 3-kinase is activated by hypoxia, or there is increased mTORC2 activation, these all result in activation of Akt which can activate mTORC1 [107]. Increased mTORC1 activity is then associated with suppression of autophagy to promote cell survival [98], alongside increased Notch3 signalling which is also pro-proliferative and pro-survival as evidenced in this review.

HIPPO signalling is a tumour suppressor pathway inactivated in many types of cancer, with reduced HIPPO signalling promoting tumour growth and survival [108]. Large tumour suppressor 1 (LATS1) is central to the HIPPO signalling pathway and is important in reducing the abundance of yes-associated protein (YAP), which promotes increased proliferation and reduced apoptosis [109]. LATS1 and therefore HIPPO signalling were found to be inactivated in small remodelled pulmonary arteries and distal PASMCs from iPAH patients [110]. This was associated with up-regulation of pro-oncogenic YAP and accumulation of NICD3 [110]. NICD and downstream HIPPO effector YAP have previously been shown to physically and functionally interact [111], suggesting integration of these two pro-proliferative pathways.

This convergence of cancer-related pathways with the Notch pathway provides additional mechanism whereby Notch3 can contribute to the proliferation and survival of pulmonary vascular cells in PAH.

### Notch3 and calcium in PASMCs

An increase in cytosolic Ca^2+^ in PASMCs is a major trigger of vasoconstriction and acts as an important stimulus for PASMC proliferation and migration [112]. Increased pulmonary vascular resistance and PASMC proliferation/migration are key processes in pulmonary vascular remodelling. Store-operated Ca^2+^ channels (SOCs) such as transient receptor potential channels (TRPCs), STIM1/2 and Orai1, mediate Ca^2+^ influx to redress cytosolic Ca^2+^ store levels through store-operated calcium entry (SOCE) [113]. Expression of SOCs is increased in hypoxic PH and is associated with increased SOCE and proliferation, [54,114]. Jag1 stimulation of human PASMC significantly increases the SOCE amplitude over a 15–30-min period, which corresponds with the time course of Jag-1-mediated increase in NICD protein level, an effect that was reduced with inhibition of Notch cleavage [115]. Hypoxia also increases SOCE in hPASMCs through a Notch-dependent mechanism which specifically affects TRP6 [75]. Treatment of chronically hypoxic mice with 2-APB, a TRP inhibitor, reduced right ventricular systolic pressure (RVSP), as did genetic deletion of *Trp6* [75].

### Potential role of non-canonical Notch3 signalling in the pulmonary vasculature

While there is growing evidence that the classical Notch3 signalling pathway plays a role in regulating pulmonary vessel function, little is known about non-canonical Notch signalling in this vascular bed. A key potential non-canonical pathway we will focus on is the emerging idea of cross-talk between the female sex hormones (oestrogens) and Notch. This is of relevance in PAH given it occurs more frequently in female patients.

Although there is currently no evidence of interplay between Notch3 and oestrogen signalling in PAH, previous studies have shown non-canonical signalling between Notch3 and oestrogens in other diseases including cancer. This is a particularly interesting area for exploration in PAH, given the sex disparity seen in PAH towards females [116] and the evidence suggesting a potentially pathological role for endogenous oestrogens in the development of the disease [117–120]. As previously highlighted several key processes involved in PAH are also driving forces in cancers [121].

It has been demonstrated that overexpression of NICD3 can transactivate oestrogen receptor (ER) α (ERα) in breast cancer cells through binding at RBP-Jκ responsive binding elements in the ERα promoter [122] and Notch1 can induce ERα-dependent transcription via IKKα-dependent recruitment of Notch transcriptional complexes [26]. There is also evidence of cross-talk in the opposite direction, in which oestrogens can activate Notch signalling in cancer cells. Oestrogen stimulation induces proliferation in both ER-positive and ER-negative endometrial cancer; however, oestrogen only activated Notch signalling and increased Notch1 expression in ER-positive cells [123]. This effect was inhibited through siRNA-mediated knockdown of GPR30, the ER thought to facilitate non-genomic oestrogen signalling, and was partially inhibited by Notch inhibition, suggesting a GPR30-Notch1 signalling axis in proliferation in these cells [123].

17β-estradiol (E2) stimulation has also been demonstrated as a modulator of Notch signalling. E2 can induce Jag1 and Notch1 expression in breast cancer MCF7 cells; increased Jag1 is abrogated by ER antagonist treatment, suggesting an ER-dependent mechanism corroborated by the discovery of imperfect oestrogen-responsive elements in the 5′ region of Notch1 and Jagged1 genes [124]. Treatment with E2 is also associated with activation of Notch signalling in Notch1 reporter gene and co-culture assays [124]. In human umbilical vein endothelial cells (HUVECs) E2 induces Notch1 signalling and under conditions where Notch is activated by Dll4 following VEGF-A treatment, E2 further increases the active form of Notch1 with increased expression of Hey2 mRNA [125]. This effect was abrogated with ER antagonism, suggesting E2 modulation of Notch1 occurs through ER signalling [125].

More recently, a role for ERβ and Notch has been proposed in the endothelium. Fortini et al. [126] described that E2 counteracts TNFα-induced apoptosis in endothelial cells via a Notch-dependent mechanism, and that this effect of oestrogen is mediated by ERβ and not ERα. Given the importance of apoptosis resistance in PAH, this signalling cross-talk could be functional in pulmonary vascular endothelium and potentially contribute to the sex-specific effects of the disease.

Investigation of an oestrogen/Notch signalling axis in the pulmonary vasculature could be interesting in the context of the remodelling seen in PAH and should be studied as a potential mechanism for the increased prevalence of women with the disease. While there is evidence of Notch1 interaction with oestrogens, a role for Notch3, shown to be expressed specifically in VSMC and with a role in PAH, and oestrogens remains to be explored.

### Notch3 and the right ventricle

While research relating to Notch3 and PAH has focussed primarily on the pulmonary circulation, there is some evidence that Notch3 in the myocardial vascular bed also plays an important role in pressure adaptation, an important process in the development of PAH.

Gene and protein expression of Notch3 is increased in the RV in mice after treatment with MCT, a known inducer of PAH and right heart failure [127]. Recent data suggest that Notch3 signalling may play a role in adaptive responses to pressure overload. *Notch3*^−/−^ mice exhibit higher mortality in response to angiotensin II-induced systemic hypertension and this is reported to be due to an increase in heart failure [74]. When challenged with angiotensin II *Notch3^−/−^* mice exhibit oedema and dilated hearts [74], and exhibit early onset acute decompensated heart failure [128]. This proposes the adaptive capability of the heart to respond to increased pressure is affected by loss of Notch3. Additionally, the same effects were seen in a smooth muscle-specific RBP-Jκ knockout animal [128]. These findings suggest that absence of canonical Notch3 signalling in normal VSMCs promotes development of heart failure in response to pressure overload, and pressure overload is an important feature in later stage PAH.

## Notch3 inhibition as a potential therapeutic target in pulmonary vascular remodelling

Understanding the role of Notch3 in pulmonary vascular remodelling will provide valuable insight into potential new treatments for PAH, a disease that is suboptimally treated. With the evidence produced to date on Notch3 in PAH, inhibition of Notch3 signalling may provide a useful therapeutic target. The viability and fertility of *Notch3*-deficient mice suggests Notch3-selective inhibitors should be well tolerated.

### γ secretase inhibitor molecules in PAH

Cleavage of the NICD by γ-secretase provides an obvious target for pharmacological blockade of Notch signalling in PAH. Research using GSIs in PAH models has focussed on N-[N-(3,5-difluorophenacetyl)-l-alanyl]-S-phenylglycine t-butyl ester (DAPT), a repurposed Alzheimer’s drug previously shown to inhibit Notch cleavage *in vivo* [129]. DAPT reduces pathological processes in various models of experimental PH.

In chronically hypoxic (10% O_2_) mice, DAPT treatment prevented development of hypoxia-induced [11]. DAPT-treated hypoxic mice also showed an intact distal pulmonary tree with normal morphology compared with the vascular pruning seen in untreated hypoxic mice [11]. These processes in DAPT-treated mice were associated with decreased Notch3 activity, and reduced PCNA expression and VSMC proliferation [11]. The same beneficial effects were seen with *in vivo* prophylactic DAPT administration to chronically hypoxic neonatal rats and adult mice; prevention of PH and associated pulmonary vascular remodelling [73,74]. Effects of DAPT have also been investigated in the MCT rat model of PH. As in the hypoxic mouse, DAPT administration significantly reduced the muscularisation of pulmonary arteries and prevented development of PH [78].

Propylthiouracil (PTU), another GSI, has also been studied *in vivo* in the MCT rat model of PAH with similar results. Daily treatment with PTU significantly reduced pulmonary artery medial thickening in MCT-treated rats and reduced expression of Notch3 in pulmonary arteries [130]. Immunocytochemistry highlighted reduced nuclear NICD3 in MCT-rat cell treated with PTU, indicating reduced nuclear translocation of the NICD after PTU treatment. [130]. Inhibition of Notch3 by PTU was found to occur through suppression of the regulatory presenilin enhancer 2 subunit within γ-secretase [130].

The success of GSIs in PAH models suggests potential for these drugs as a new therapeutic approach in human PAH. This class of drugs is already under investigation in human trials for the treatment of certain cancers where Notch signalling is unregulated [131].

### Inhibition of Notch3 with endogenous molecules

Some endogenous molecules have been described as inhibitors of Notch3 signalling, and these may offer advantages over exogenous drugs. Notch3 receptors can be stimulated by multivalent or monovalent ligands. Previous studies suggested that monovalent soluble Jagged1 (sJag1) inhibits Notch signalling [132], while others showed that endogenous cell-bound ligands increase Notch signalling. Endogenous miRs have also been shown to interact with and inhibit the activity of Notch3. miR-206 was identified originally as a tumour-suppressor molecule. Overexpression of miR-206 reduces tumour cell proliferation in hepatocellular carcinoma [133] and colorectal cancer [134] cell lines. With the similarities in cell behaviour seen in both cancers and PAH, interest in miRs in PAH has grown. The effects of miR-206 on Notch3 signalling in cancer cells translates to pulmonary vascular cells. In PASMCs from chronically hypoxic mice miR-206 is down-regulated, and this was inversely correlated with a significant increase in Notch3 and cardiac indices of PH [135]. Transfection of human pulmonary microvascular endothelial cells (hMPECs) with miR-206 mimic [136], and transfection of hPASMCs and mPASMCs from chronically hypoxic mice [135] with miR-206 plasmid to induce overexpression, promotes apoptosis. In hMPECs this effect is abrogated by overexpression of Notch3 [136]. Overexpression of miR-206 in hPASMCs also increased expression of vascular smooth muscle differentiation markers calponin and α-SMA, while reducing Notch3 expression, migration and proliferation [135]. These results suggest down-regulation of miR-206 in PAH may contribute to Notch3 up-regulation and the pro-proliferative/anti-apoptotic phenotype observed in patients. Therapeutic use of other miRs, such as anti-miRNAs, is already being investigated in PH models [137,138]. *In vivo* use of molecules such as miR-206 in models could soon follow.

### Monoclonal antibody targeting of Notch3

While Notch inhibition by GSIs and ligands may be beneficial in PAH models, these drugs are not specific to any one Notch receptor subtype, and pan-inhibition of all Notches can have many off-target side effects that may hamper their use in patients. One solution has been the introduction of therapeutic monoclonal antibodies specific to Notch3, providing a strategy to down-regulate activity of Notch3. Monoclonal Notch3 antibody treatment has already been tested in experimental models of CADASIL, where treatment was shown to improve cerebrovascular function in the TgN3^R169C^ CADASIL mouse model [139], which has a gain of function of Notch3 [140]. Studies are now exploring potential therapeutic use of monoclonal antibodies for PAH. Gower et al. [141] developed a humanised monoclonal Notch3 antibody that binds to the Jag1-binding site on the murine and human receptors, blocking ligand binding and hence reducing cleavage and downstream signalling. *In vivo*, this antibody has shown promising results by reducing parameters of established PAH in both chronic-hypoxic mouse and Sugen-hypoxic rat models, with concurrent reduction in levels of cleaved Notch3 in treated animals, suggesting antibody therapy had a direct effect on Notch3 downstream signalling [141].

## Conclusions

The evidence highlighted in this review demonstrates a clear involvement of VSMC Notch3 in pulmonary vascular remodelling in PAH. However, many of the specific signalling pathways and molecular mechanisms are still unknown. It will be important to examine the role of Notch dynamics to determine whether specific effectors involved in Notch3 signalling in pulmonary cells have distinct functional effects in the pulmonary vasculature. Furthermore, while non-canonical Notch signalling has been investigated in other conditions, there is a paucity of information regarding its potential role in PAH and vascular remodelling and this area could hold major answers to Notch3-mediated mechanisms involved in PAH progression. We have also described several Notch3-specific avenues of intervention currently being explored. However, further preclinical and clinical investigations are needed to develop these approaches, pinpoint more useful therapeutic targets, and translate these into potential new treatments for PAH.

## Abbreviations

ADAM: a disintegrin and metalloproteinase
2-APB: 2-Aminoethoxydiphenyl borate
Bcl2: B-cell lymphoma 2
BCL2L1: Bcl-2-like protein 1
Bcl-xL: B-cell lymphoma-extra large
BMP: Bone Morphogenic Protein
CADASIL: cerebral autosomal dominant arteriopathy with sub-cortical infarcts and leukoencephalopathy
c-FLIP: FADD-like IL-1β-converting enzyme)-inhibitory protein
DAPT: N-[N-(3,5-Difluorophenacetyl)-l-alanyl]-S-phenylglycine t-butyl ester
Dll: Delta-like protein
E2: 17β-estradiol
EGF: Epidermal G
ER: oestrogen receptor
GPR30: G protein-coupled receptor 30
GSI: γ-secretase inhibitor
Hes: Hairy and enhancer of split
Hey: Hairy/enhancer-of-split related with YRPW motif
HIF-1α: hypoxia inducible factor 1
hMPEC: human pulmonary microvascular endothelial cell
hPASMC: human pulmonary artery smooth muscle cell
hPAH: human pulmonary arterial
IKKa: IκB kinase alpha
IPAH: Idiopathic pulmonary arterial hypertension
Jag: Jagged protein
KLF: Krüppel-like factor
LATS1: large tumour suppressor 1
MCT: monocrotaline
MHC: major histocompatibility complex
miR: microRNA
mTOR: mammalian target of rapamycin
mTORC: mTOR complex
NECD: Notch extracellular domain
NICD: Notch intracellular domain
PAH/PH: pulmonary arterial hypertension/pulmonary hypertension
PARP-1: poly (ADP-ribose) polymerase 1
PASMC: pulmonary artery smooth muscle cell
PCNA: Proliferating cell nuclear antigen
PTU: propylthiouracil
SMA: smooth muscle actin
SOC: store-operated Ca^2+^ channel
SOCE: store-operated calcium entry
TCTP: translationally controlled tumour protein
VEGFA: Vascular endothelial growth factor A
VSMC: vascular smooth muscle cell
YAP: yes-associated protein
